# Attitudes of specialist memory-clinic patients with early symptomatic Alzheimer’s disease towards lecanemab: results from a multicenter survey in Europe

**DOI:** 10.1038/s41598-026-61640-1

**Published:** 2026-07-09

**Authors:** Jonathan Vöglein, Johannes Levin, Elisabeth Stögmann, Christian Haass, Günter U. Höglinger, Lutz Frölich, Frank Jessen

**Affiliations:** 1 Department of Neurology, LMU University Hospital, LMU Munich, Marchioninistraße 15, 81377 Munich, Germany; 2 German Center for Neurodegenerative Diseases (DZNE), Feodor-Lynen-Straße 17, 81377 Munich, Germany; 3 Munich Cluster for Systems Neurology (SyNergy), Feodor-Lynen-Straße 17, 81377 Munich, Germany; 4 Deutsches Netzwerk Gedächtnisambulanzen, Pauwelsstraße 30, 52074 Aachen, Germany; 5 Department of Neurology, Medical University of Vienna, Währinger Gürtel 18-20, 1090 Vienna, Austria; 6 Comprehensive Center for Clinical Neurosciences and Mental Health, Medical University of Vienna, Währinger Gürtel 18-20, 1090 Vienna, Austria; 7 Biomedicine Center (BMC), Biochemistry, LMU Munich, Großhaderner Straße 9, 82152 Planegg, Germany; 8 Department of Geriatric Psychiatry, Central Institute of Mental Health Mannheim, Medical Faculty Mannheim, University of Heidelberg, Heidelberg, J5, 68159 Mannheim, Germany; 9 European Alzheimer’s Disease Consortium, Kerpener Straße 62, 50931 Cologne, Germany; 10 Department of Psychiatry, University of Cologne, Medical Faculty, Kerpener Straße 62, 50931 Cologne, Germany; 11 German Center for Neurodegenerative Diseases (DZNE), Bonn/Cologne, Venusberg-Campus 1, 53127 Bonn, Germany

**Keywords:** Alzheimer’s disease, Lecanemab, Survey, Patient attitudes, Specialist memory clinic, Diseases, Medical research, Neurology, Neuroscience

## Abstract

Lecanemab approval in the European Union (EU) was granted after a delay. This delay resulted in concerns from many stakeholders, but attitudes of patients with early symptomatic Alzheimer’s disease receiving specialist memory-clinic care remained insufficiently assessed. Therefore, we evaluated attitudes of specialist memory-clinic patients with early symptomatic Alzheimer’s disease towards lecanemab in Europe. In this anonymous, international, multicentre, cross-sectional survey conducted from October 14, 2024 to February 18, 2025, a standardized, expert-developed questionnaire assessed attitudes towards lecanemab treatment and EU approval. Before answering four binary questions, participants received brief explanatory information on expected clinical benefit, amyloid-related imaging abnormalities (ARIA), and the increased ARIA risk associated with APOE ε4 homozygosity. The survey was conducted in specialist memory clinics within the European Alzheimer’s Disease Consortium (EADC), the German memory clinic network (DNG), and Austrian memory centers. Available recruitment-flow data were limited to completed questionnaires because the survey was anonymous and distributed locally. 281 patients with early symptomatic Alzheimer’s disease completed the survey. Network-level sample sizes were EADC n = 202, DNG n = 60, and Austria n = 19; country-specific sample sizes within the EADC and response rates were not available. Endorsement was high for both individual treatment with lecanemab (81.9%, 95% confidence interval [CI] 76.8–86.2) and general EU approval (91.8%, 95% CI 87.9–94.7). Endorsement remained substantial, but was lower, in the context of APOE ε4 homozygosity (treatment: 61.2%, 95% CI 55.2–66.9; approval: 76.5%, 95% CI 71.1–81.3). Approval-related questions received higher endorsement than treatment-related questions (84% vs. 72%; *p* < 0.001). Support for approval for APOE ε4 homozygotes declined after regulatory recommendations excluded this group (from 87% to 73%; *p* = 0.025); this comparison reflects independent respondents completing the anonymous survey before versus after November 14, 2024. Network-level comparisons were descriptive and underpowered for geographic inference. High endorsement within this specialist memory-clinic sample suggests perceived value of access to lecanemab. Greater endorsement for approval than for individual treatment may reflect support for treatment access beyond personal treatment choice, but alternative explanations such as social desirability, acquiescence, misunderstanding, or effects of the survey information cannot be excluded. The findings should not be generalized beyond specialist memory-clinic patients and should be interpreted in view of potential selection and response biases, absent response-rate data, and the brief, non-validated binary questionnaire.

**Supplementary Information:**

The online version contains supplementary material available at 10.1038/s41598-026-61640-1.

## Introduction

Lecanemab is a monoclonal antibody directed against amyloid-β that effectively clears amyloid-β plaques from the brain^1^. In a phase 3 trial, lecanemab slowed down clinical progression of Alzheimer’s disease (AD) by about 30% in 18 months^1^. As a result, lecanemab was approved for treatment of early symptomatic Alzheimer’s disease in several regions worldwide, including the USA^2^, Japan^3^, China^4^, South Korea^5^, Hong Kong^6^, Israel^7^, the United Arab Emirates^8^ and the UK^9^, and, after a prolonged regulatory process, in the European Union (EU)^10^. In Europe, the course of this evaluation process was highly visible and debated, and has sparked several position statements. Various stakeholder groups, including scientists^11,12^, scientific journals^13^, medical and scientific societies^11,14,15^, publicly funded research organisations^16^, patient advocacy groups^17–19^, the manufacturer, and the media^21,22^, voiced concerns or difficulty understanding the initial negative CHMP opinion.

Treatment with lecanemab can be associated with amyloid related imaging abnormalities (ARIA), a class-specific side effect of monoclonal amyloid-β antibodies. In the phase 3 trial of lecanemab, ARIA occurred in approximately 12% of treated patients^1^. In one quarter of these 12%, i.e. in about 3% of all patients treated with lecanemab, ARIA were accompanied by symptoms. Whereas no deaths associated with lecanemab occurred in the core phase 3 trial, in the open-label extension phase ARIA were associated with fatal outcomes in isolated cases^23^. In an opinion of 25 July 2024, the Committee for Medicinal Products for Human Use (CHMP) of the European Medicines Agency (EMA) considered the benefit/risk-ratio of lecanemab not sufficient and recommended against an approval of the drug in the European Union (EU)^24^. After a re-examination procedure, the CHMP adopted a positive opinion published on November 14, 2024. Similar to the UK, this positive opinion restricted eligibility to individuals who are not homozygous for APOE ε4^24^. After a second re-examination considering newly available safety data, the CHMP concluded that its November opinion did not require revision. Consequently, the European Commission resumed the decision-making procedure regarding the market authorization of lecanemab^24^. Finally, EU approval was granted on April 15, 2025^10^.

However, knowledge about the attitudes toward lecanemab of one key stakeholder group was missing: specialist memory-clinic patients with early symptomatic AD who are potentially eligible for lecanemab treatment. To address this gap, and to provide the direct perspective of this clinic-based patient group during a period of active European regulatory debate, we conducted a cross-national survey across specialist memory clinics in Europe.

## Methods

### Participants

Patients with mild cognitive impairment or mild dementia and a confirmed positive amyloid-β status, that is, patients with early symptomatic AD potentially suitable for treatment with lecanemab, were eligible to participate in the survey. Eligibility was determined by treating neurologists and/or psychiatrists. There were no additional clinical exclusion criteria at the analysis stage; only complete questionnaires were analyzed, and the survey required answers to all four items.

The survey was conducted anonymously and collected no personal, demographic, site-level, or country-level data. All methods were carried out in accordance with relevant guidelines and regulations. The ethics committee of LMU Munich reviewed the study (reference number: 24-0894-KB) and confirmed that no formal ethics approval was required and no individual written informed consent was needed for this fully anonymous survey.

### Survey design

The primary goal for the survey design was to convey information about the benefits and risks of lecanemab as simply as possible, using lay language appropriate for cognitively impaired individuals, while ensuring sufficient detail to enable well-informed responses. The survey was designed by JV, FJ, and LF, with advisory input from JL. The design process involved both email correspondence and several in-person meetings. Before being asked to answer four yes/no questions, participants received a brief standardized introductory text describing progressive clinical decline in AD, an approximate 30% slowing of progression over 18 months with lecanemab, ARIA frequency and symptom risk, the potential need for repeated MRI monitoring in the event of ARIA, and the occurrence of rare severe events. A separate question-specific text explained that ARIA risk is higher in APOE ε4 homozygotes. The exact wording is shown in Supplementary Figs. 1 and 2. The survey was administered using the online platform SurveyMonkey. No formal psychometric validation or pilot study was performed; the questionnaire should therefore be understood as an expert-developed instrument rather than a formally validated patient-reported measure.

### Survey distribution

The survey was distributed through memory clinics of the German Network of Memory Clinics (Deutsches Netzwerk Gedächtnisambulanzen, DNG)^25^, memory clinics in Austria affiliated with the departments of neurology at medical universities in Vienna, Graz, Innsbruck and Salzburg, and within the European Alzheimer’s Disease Consortium (EADC)^26^. The DNG and the EADC are non-profit medical and scientific organizations. The DNG provides a national network for AD centers in Germany^25^, while the EADC promotes collaborative AD research across multiple European countries^26^. Standardized versions in German language were used for Germany and Austria, and a standardized English version for the EADC. After eligible patients were identified by their treating physicians, a survey link was provided. For patients lacking the necessary technical skills or resources to complete the online survey, a paper version could be filled out manually and later entered into the online system by the treating physician. For patients not fluent in English, EADC centers had the option to translate the English survey version into local languages using an artificial intelligence-based translation tool. Because the link was distributed locally by treating physicians and the survey was fully anonymous, the number of eligible patients approached or invited, non-participants, and incomplete attempts could not be reconstructed; therefore, response rates were unavailable. Country-specific sample sizes within the EADC were also unavailable. The survey was launched on October 14, 2024, in the DNG, and on October 16, 2024, in Austria and the EADC. Common closing was on February 18, 2025.

### Statistical analysis

The main results are reported descriptively as proportions with 95% confidence intervals (CIs).

Fisher’s exact test was used to examine the influence of question type (treatment- vs. approval-related questions), population context (general vs. APOE ε4 homozygosity), network-defined population (DNG, Austrian centers, EADC) and timepoint of survey completion (before vs. after CHMP’s positive opinion on November 14 2024) on responses. Because the survey was anonymous and cross-sectional, the before-versus-after comparison reflects independent response sets by calendar period rather than repeated measurements in the same individuals. Fisher’s exact test was chosen over regression models because it is more appropriate for small or imbalanced subgroup sizes and handles complete separation (e.g., 100% endorsement for lecanemab approval for APOE ε4 homozygotes in Austria) without instability. The network-defined subgroup analyses were interpreted descriptively because the Austrian subgroup was small and country-specific sample sizes within the EADC were unavailable. All tests were two-sided. *p*-values < 0.05 were considered statistically significant. Statistical analyses were conducted using R version 4.3.3.

## Results

### Survey design and language

The appearance and wording of the survey are shown in supplementary Figs. 1 and 2. In brief, participants were asked to read a short explanatory text summarizing the expected clinical benefit of lecanemab, the frequency and possible clinical consequences of ARIA including monthly MRI monitoring, and severe adverse events in some patients. Subsequently, four yes/no questions addressing the following topics were asked: 1. the wish to be treated with lecanemab; 2. the opinion on the approval of lecanemab in the EU; 3. the wish to be treated with lecanemab in the hypothetical case of APOE ε4 homozygosity; 4. the opinion on the approval of lecanemab for APOE ε4 homozygotes in the EU.

### Participants

A total of 281 patients with early symptomatic AD completed the questionnaire. Network-level sample sizes were 202 patients (72%) from EADC-affiliated sites, 60 (21%) from DNG centers in Germany, and 19 (7%) from memory clinics in Austria. The EADC population included, but was not limited to, responses from Belgium, Bulgaria, Czech Republic, Greece, France, Italy, Portugal, Slovakia, Spain and Sweden. Country-specific sample sizes within the EADC were not collected.

This constitutes the available participant-flow information. Because the survey was anonymous and distributed locally, the number of eligible or invited patients, non-participants, and incomplete attempts was not available; thus, response rates could not be calculated. Because all four questions had to be answered to complete the questionnaire, there were no item-level missing data among completed surveys.

### Survey results

**Fig. 1:**
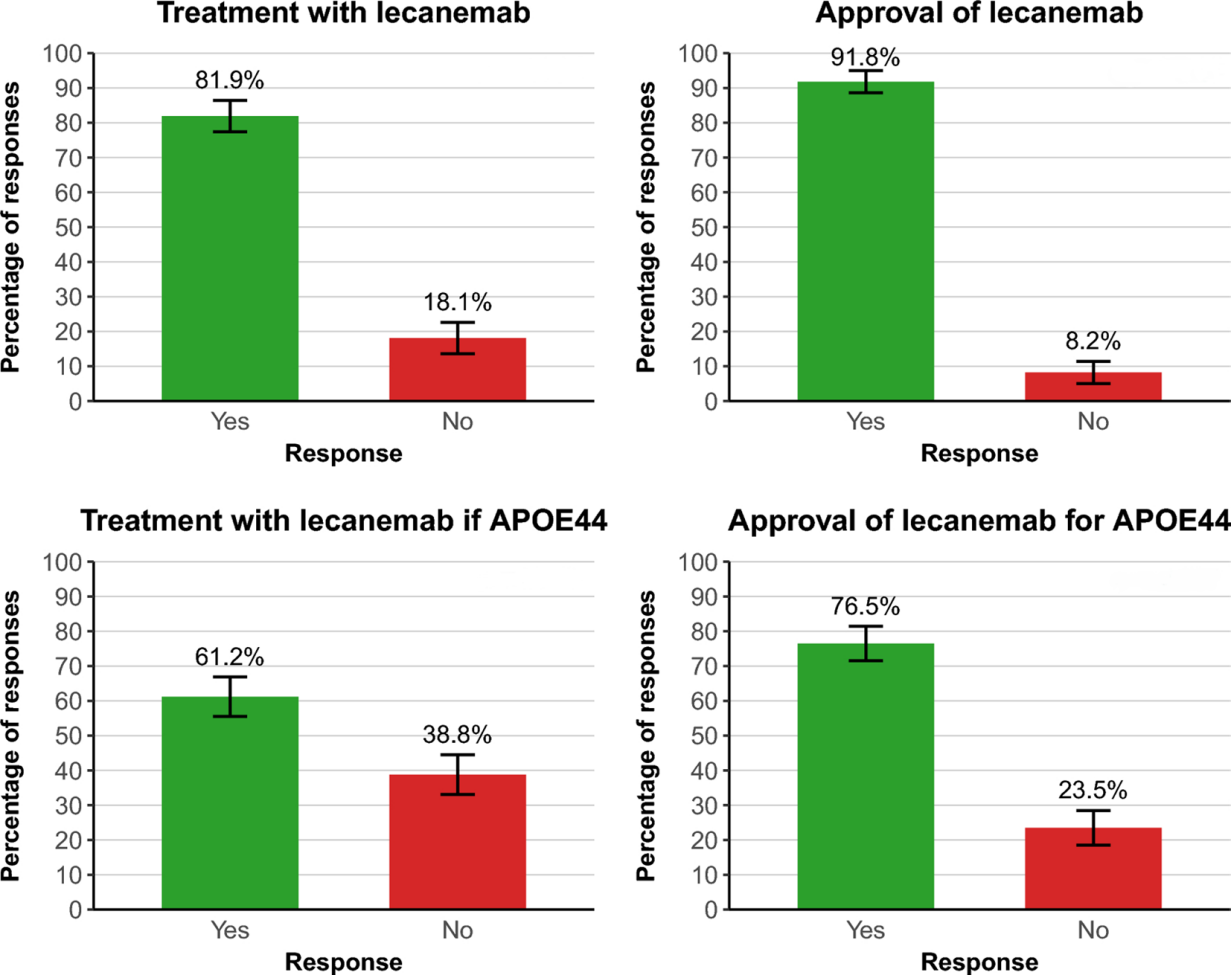
Survey responses regarding lecanemab treatment and regulatory approval. Description: Panel titles reflect the topics addressed in the corresponding survey questions. The majority of respondents with early symptomatic Alzheimer’s disease supported individual treatment with lecanemab and its approval in the European Union (EU). While endorsement remained high, it was lower for treatment willingness in case of APOE ε4 homozygosity and for EU approval for APOE ε4 homozygotes. Error bars indicate 95% confidence intervals. APOE44, APOE ε4 homozygous/APOE ε4 homozygotes.

A total of 81.9% of survey participants indicated that they would wish to receive lecanemab if it were available (230/281; CI: 76.8–86.2%). Endorsement of EU approval of lecanemab was higher, with 91.8% in favor (258/281; CI: 87.9–94.7%). When asked about treatment preference in the case of known APOE ε4 homozygosity, 61.2% expressed a wish to be treated (172/281; CI: 55.2–66.9%). Approval of lecanemab for APOE ε4 homozygous AD patients was endorsed by 76.5% of respondents (215/281; CI: 71.1–81.3%) (Fig. 1, Supplementary Table).

Approval-related questions received higher endorsement than treatment-related ones, and questions framed in the general population context were endorsed more than those targeting APOE ε4 homozygotes.

### Response patterns

To explore how individual participants responded across all four survey questions, response combinations were analyzed. The most frequent pattern, endorsed by 56.6% of respondents, included affirmative answers to all four questions. Other response patterns were markedly less common. For example, 11.7% of participants supported approval for all early AD patients, including APOE ε4 homozygotes, and wished to be treated with lecanemab in the case they were APOE ε4/ε4 non-carriers, but not if they were hypothetically APOE ε4 homozygous. Complete non-endorsement across all four items was rare and observed in only 6.8% of participants.

**Fig. 2:**
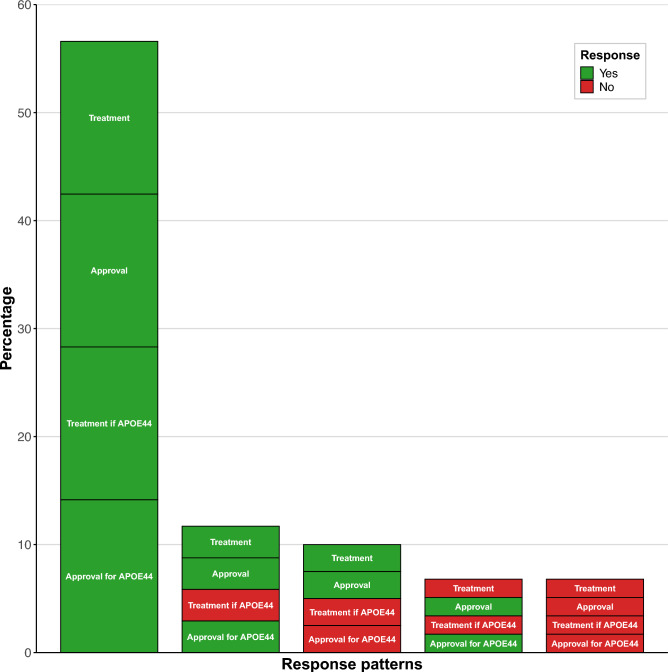
Survey response patterns. Percentages of the top five response combinations observed in the study are shown. The most frequent pattern by far (56.6% of participants) was full endorsement of both individual treatment and regulatory approval, in both general and APOE ε4 homozygosity-specific contexts. APOE44, APOE ε4 homozygous/APOE ε4 homozygotes.

Patterns with mixed responses confirmed the stronger endorsement of approval questions over treatment questions, and of general population items over those targeting APOE ε4 homozygosity questions. Figure 2 illustrates the response combinations observed in more than 5% of participants.

### Survey outcomes by question type (treatment vs. approval)

**Fig. 3:**
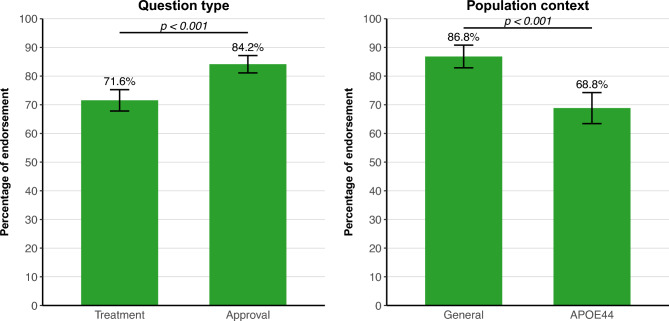
Endorsement by question type and population context. Endorsement rates were significantly higher for questions related to approval compared to those addressing treatment (left panel). Questions referring to the general early Alzheimer’s disease population received higher endorsement than those referring to APOE ε4 homozygotes (right panel). Error bars indicate 95% confidence intervals. APOE44, APOE ε4 homozygosity.

To further investigate the observed trend of higher endorsement of approval-related compared to treatment-related questions, we grouped questions by targeted topic and conducted statistical testing. Endorsement for approval questions was statistically significantly more frequent (general context (irrespective of APOE status): 92%; APOE ε4 homozygosity context: 77%) than for those addressing treatment (general context: 82%; APOE ε4 homozygosity context: 61%) (odds ratio [OR] = 2.48, 95% CI [1.44, 4.40], *p* < 0.001) (Fig. 3, left panel).

### Results by population context (general vs. APOE ε4 homozygosity)

Building on the observed pattern of higher endorsement in the general population context compared to the APOE ε4 homozygosity context, we categorized questions accordingly and carried out statistical analysis. Although endorsement rates were generally high, endorsement was statistically significantly higher for questions referring to the general early AD population context (approval: 92%; treatment: 82%) than for those referring specifically to APOE ε4 homozygosity (approval: 77%; treatment: 61%) (OR = 2.98, 95% CI [2.18, 4.09], *p* < 0.001) (Fig. 3, right panel).

### Survey findings by network-defined population (DNG, Austria, EADC)

**Fig. 4:**
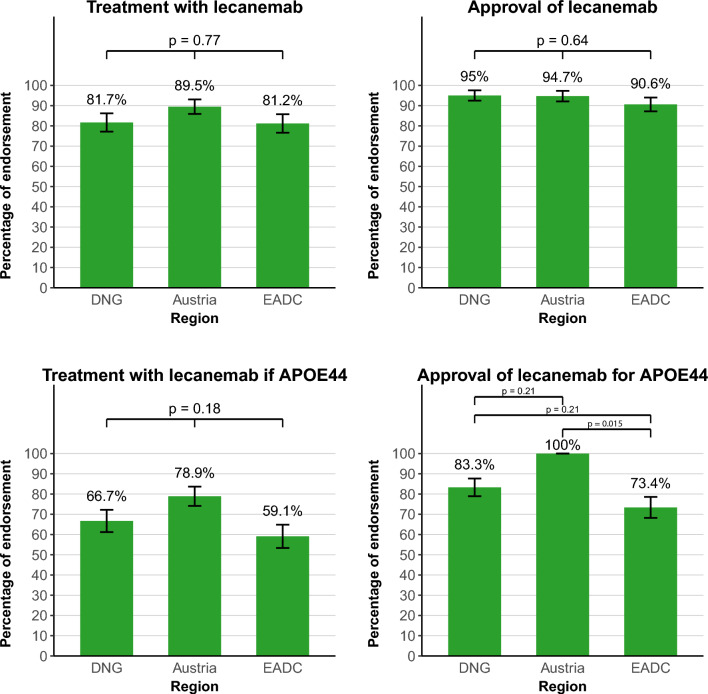
Endorsement by network-defined population. Endorsement did not significantly differ across DNG, Austrian, and EADC respondents for three of the four survey questions. For the question addressing approval for APOE ε4 homozygotes, a statistically significant difference was observed between Austria and EADC, potentially driven by complete response separation and the smaller sample size in the Austria population. These comparisons were descriptive and underpowered for geographic inference. Error bars indicate 95% confidence intervals. APOE44, APOE ε4 homozygous/APOE ε4 homozygotes; DNG, Deutsches Netzwerk Gedächtnisambulanzen (German Network of Memory Clinics); EADC, European Alzheimer’s Disease Consortium.

When examining endorsement by network-defined population, the descriptive patterns were broadly similar, but these comparisons should be interpreted cautiously because the Austrian sample was small (n = 19) and country-specific EADC sample sizes were unavailable. A nominal statistically significant difference was found for the APOE ε4 homozygosity-specific approval question (*p* = 0.007). Post hoc pairwise comparisons (Holm-adjusted) indicated that this effect was primarily driven by a difference between Austria and EADC (*p* = 0.015), while other comparisons (DNG vs. Austria, DNG vs. EADC) were not significant after correction (*p*-values = 0.21). No significant differences between network-defined populations were observed for the other three questions (*p*-values > 0.17) (Fig. 4, Supplementary Table).

### Responses by timepoint (before vs. after CHMP’s positive opinion)

**Fig. 5:**
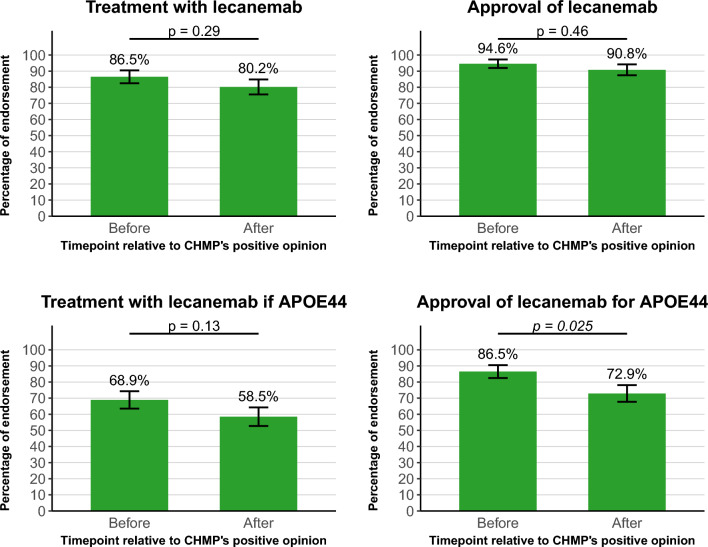
Endorsement relative to CHMP’s positive opinion regarding lecanemab approval in the European Union. For the approval question specific to APOE ε4 homozygotes (bottom right panel), endorsement was significantly lower after CHMP’s opinion (which excluded APOE ε4 homozygotes from the proposed label) than before. No significant differences were observed for the remaining three questions. Error bars indicate 95% confidence intervals. APOE44, APOE ε4 homozygous/APOE ε4 homozygotes; CHMP, Committee for Medicinal Products for Human Use.

When stratified by timepoint (before vs. after the CHMP’s positive opinion), for the APOE ε4 homozygosity-specific approval question, endorsement was significantly lower after CHMP’s opinion (that excluded APOE ε4 homozygotes from approval) than before (before: 86.5%; after: 72.9%) (*p* = 0.025, OR = 0.42, 95% CI [0.18, 0.90]). No statistically significant differences were observed for the remaining three questions (treatment before vs. after: 86.5% vs. 80.2%; approval: 94.6% vs. 90.8%; treatment if APOE ε4 homozygous: 68.9% vs. 58.5%; all *p*-values > 0.12) (Fig. 5).

## Discussion

In this survey of specialist memory-clinic patients with early symptomatic AD in Europe, high endorsement was observed for lecanemab as both a treatment option and as a drug candidate for regulatory approval. Endorsement exceeded 50% for all four survey questions. Notably, more than 90% of participants supported lecanemab’s approval in the EU, and over 80% indicated a personal wish to receive treatment with the drug. These findings provide descriptive insights into attitudes among a selected specialist-care patient group during a period when regulatory decisions were still pending in the EU^24^. They should not be interpreted as representative of all patients with AD. The high endorsement observed in this survey aligns with widespread reactions from scientific, medical and patient communities during the regulatory decision-making process, many of which expressed incomprehension at the initial negative CHMP opinion^11–22^.

In addition to the high endorsement for lecanemab use in the EU, our analysis revealed two main response patterns. First, approval-related questions consistently received higher endorsement than treatment-related questions. One possible interpretation is that some respondents distinguished personal treatment decisions from broader regulatory access: while some individuals may hesitate to pursue treatment for themselves because of safety concerns, uncertainty about benefits, or personal values, they may nonetheless support access to lecanemab for others. This interpretation is consistent with the literature on patient autonomy and shared decision-making in dementia care^27–29^, and with previous preference research showing heterogeneous benefit-risk trade-offs in future AD treatments^30^. However, the survey cannot distinguish this explanation from alternative mechanisms, including altruistic support for options for others, trust in regulators or physicians, general attitudes toward therapeutic innovation or access, social desirability, or acquiescence to information presented by treating physicians. Accordingly, the approval-versus-treatment difference should be interpreted as an attitude pattern elicited in a defined informational context rather than direct evidence of an autonomy preference.

The second response pattern was that questions framed in the context of the general AD population received higher endorsement than those referring specifically to APOE ε4 homozygotes. While the general concept of treatment or approval for early AD was met with broad endorsement, the introduction of APOE ε4 homozygosity, an established risk factor for ARIA, appears to temper endorsement. Although endorsement was lower for APOE ε4 homozygosity-specific questions, the majority of participants still endorsed treatment and approval in this subgroup. This stands in contrast with the exclusion of APOE ε4 homozygotes from treatment^9,10^ and may be consistent with some respondents accepting higher risks under certain conditions. However, because individual understanding was not formally assessed, the survey cannot determine whether this endorsement reflected fully informed risk tolerance, misunderstanding, or simplified decision-making under the survey conditions.

Notably, endorsement of approval for APOE ε4 homozygotes was not static over time. When stratified by timepoint, support for approval in this subgroup was significantly lower after the CHMP issued its opinion recommending approval for lecanemab while excluding APOE ε4 homozygotes from the proposed treatment indication. This decline, from 87 to 73%, is compatible with the possibility that regulatory decisions and communication may shape patient perceptions, potentially reinforcing or amplifying concerns about treatment risks in this genetic subgroup. However, the survey did not assess causal mechanisms, and the before-versus-after comparison involved independent respondents rather than repeated measurements in the same patients.

At the network level, endorsement patterns were broadly similar across DNG, Austrian, and EADC respondents. However, these analyses do not provide robust evidence for geographic variation or absence thereof. A statistically significant difference emerged only for the APOE ε4 homozygosity-specific approval question, where endorsement was higher in the Austrian subgroup compared to the EADC. This effect appears to have been driven by complete endorsement among Austrian respondents, combined with the small sample size of the Austria group, which may have amplified statistical contrasts. Because the Austrian sample comprised only 19 patients and country-specific EADC denominators were unavailable, regional comparisons should be regarded as descriptive and hypothesis-generating. Future studies should prospectively collect country-specific denominators and adequate sample sizes to evaluate geographic variation.

This study has several limitations that should be stated explicitly. First, the survey was conducted in specialized memory clinics within academic and expert networks; the results therefore cannot be generalized to the broader AD population, including individuals outside specialist care or those with different socioeconomic, educational, or healthcare-access backgrounds. Second, recruitment was distributed and anonymous; only completed questionnaires were available. Numbers of eligible patients, invited patients, non-participants, and incomplete survey attempts could not be reconstructed, and response rates were therefore unavailable. Country-specific sample sizes within the EADC were also unavailable. Third, the small Austrian sample (n = 19), absence of country-level EADC denominators, and imbalanced subgroup sizes mean that network/regional comparisons were underpowered and should be interpreted only descriptively. Fourth, no personal, demographic, or disease-severity data were collected. While this preserved anonymity, it prevented analyses of potentially important sources of heterogeneity, such as age, sex, education, disease stage, health literacy, or prior treatment experience. Fifth, the instrument was deliberately brief and used binary response options to maximize feasibility in a cognitively impaired population, but this also reduced granularity and did not capture dimensions such as degree of risk tolerance, uncertainty, trust in institutions, or willingness to accept treatment burden. Sixth, the questionnaire was expert-developed but not formally validated or pilot-tested, and non-English versions within the EADC could be based on local AI-assisted translations, which may have introduced variability in interpretation. Finally, participants’ understanding of the information sheet was not formally assessed. High endorsement may therefore partly reflect the persuasiveness or framing of the standardized information sheet, physician-mediated recruitment, compliance or acquiescence bias, social desirability, misunderstanding, or trust in authority figures rather than genuine informed preferences. These factors could have led to overestimation of endorsement and limit interpretation of the approval-versus-treatment difference as evidence of autonomy preference.

Strengths of the survey include its specific focus on patients with biomarker-confirmed early symptomatic Alzheimer’s disease recruited through specialized European memory-clinic networks during an active regulatory evaluation process. The inclusion of patients with biomarker-confirmed early symptomatic AD, rather than individuals who only express concerns about developing AD, restricts the survey to a population that is potentially eligible for lecanemab. The survey was conducted across multiple specialist memory-clinic networks and addressed a concrete real-world question during an active regulatory decision-making process. In this sense, the study complements more granular preference research by offering a pragmatic cross-national snapshot of attitudes in a clinically relevant specialist-care population during regulatory uncertainty.

The survey findings raise broader questions about the role of patients in regulatory decision-making processes. Beyond ethical considerations of autonomy and shared decision-making, the involvement of patients in the assessment of therapeutic value is increasingly recognized as a legal and institutional imperative^31^. The responses to the CHMP’s initial negative opinion by Alzheimer advocacy organizations and civil society stakeholders^17–19^ underscore the relevance of including lived experience in regulatory deliberation. This is also consistent with broader principles of patient engagement and stakeholder involvement, which emphasize inclusive, transparent, and participatory approaches to healthcare policy and regulatory decision-making^31^. As disease-modifying therapies for AD continue to emerge^32^, greater integration of patient perspectives may enhance the legitimacy, responsiveness, and acceptance of regulatory decisions.

Whether these findings generalize to other anti-amyloid therapies remains uncertain. Lecanemab and donanemab share a clinical context as monoclonal amyloid-β antibodies for early symptomatic AD, and donanemab demonstrated efficacy in a positive phase 3 trial^33^ and has subsequently been approved in multiple regions worldwide, including the USA^34^, Japan^35^, China^36^, and the UK^37^. Similar to the regulatory evaluation process of lecanemab, the CHMP initially issued a recommendation to refuse marketing authorization of donanemab in the EU on March 27, 2025, which was revised to a positive opinion following a re-examination on July 24, 2025^38^, and subsequently EU approval by the European Commission was granted on September 25, 2025^39^. However, the present survey did not assess donanemab specifically; therefore, transferability to other therapies should be regarded as hypothesis-generating rather than established.

In summary, this survey provides timely insights into the attitudes of specialist memory-clinic patients with early symptomatic AD in Europe toward lecanemab. The high endorsement observed within this selected sample, both for treatment and for regulatory approval, suggests that respondents valued the availability of therapeutic options, even when associated with complex risk–benefit profiles. The results further suggest that some respondents endorsed treatment and approval decisions involving genetic risk; however, the study cannot determine whether these attitudes reflected fully informed preferences, effects of the information sheet, social desirability, acquiescence, or physician-mediated selection. As regulatory agencies continue to evaluate emerging therapies for AD, these findings may underscore the importance of integrating patient perspectives into benefit–risk assessments and policy decisions. However, because of the study design, the results should be interpreted as exploratory and hypothesis-generating. Future work should build on these data using validated and more granular preference instruments, formal comprehension checks, richer clinical and demographic characterization, prospective participant-flow documentation including response rates, country-specific sampling, and broader recruitment beyond specialist memory clinics. A key unresolved question is how best to operationalize meaningful patient involvement in regulatory, clinical, and ethical frameworks surrounding disease-modifying therapies in Alzheimer’s disease.

## Supplementary Information

Supplementary Information.

## Data Availability

All data supporting the findings of this study are available within the paper and its Supplementary Information.
