# Supplementary material for: Attitudes of specialist memory-clinic patients with early symptomatic Alzheimer’s disease towards lecanemab: results from a multicenter survey in Europe
Source: Sci Rep. 2026 Jul 9;16:21414. doi: 10.1038/s41598-026-61640-1 (PMC13350719; doi:10.1038/s41598-026-61640-1)
Supplement: Supplementary file 1 — Supplementary Information. [file 41598_2026_61640_MOESM1_ESM.docx]

**Supplementary Material**


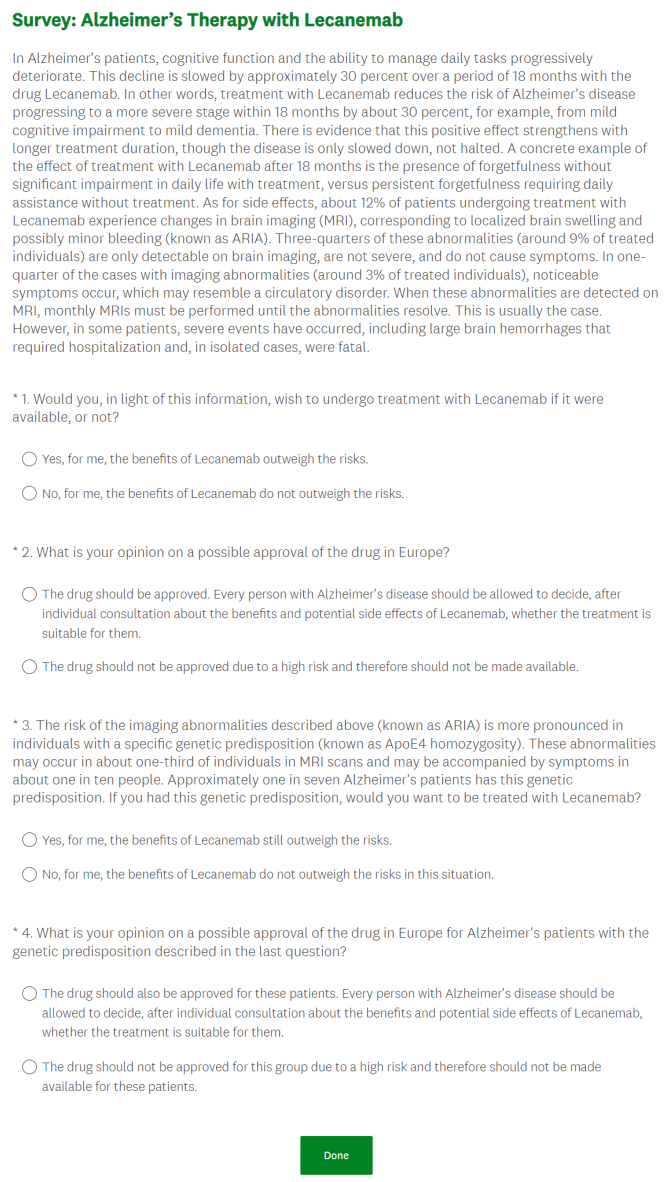


Supplementary Figure 1: **Survey appearance and wording in English.**


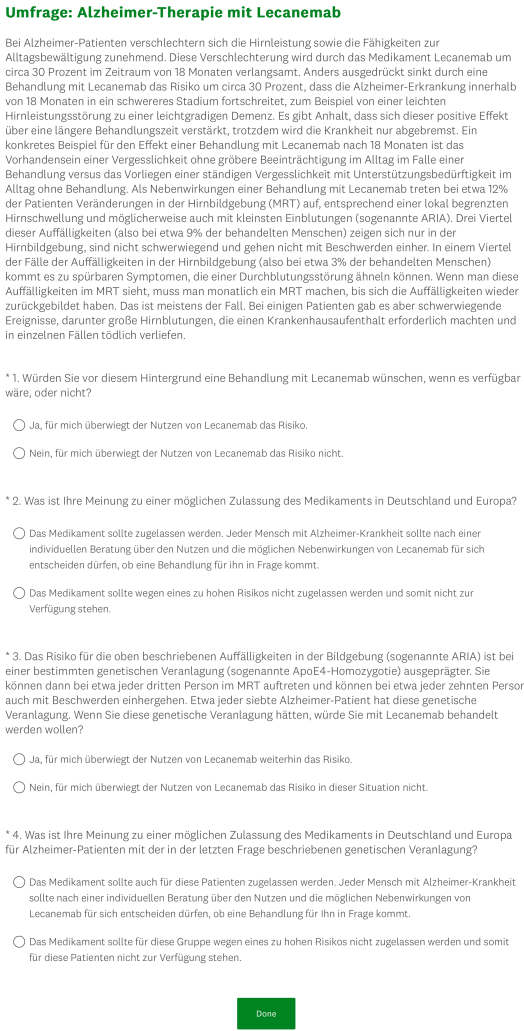


Supplementary Figure 2: **Survey appearance and wording in German.**

|  | DNG | Austria | EADC | Total Population |
| --- | --- | --- | --- | --- |
| Treatment,  yes/total responses (%) | 49/60 (81.7%) | 17/19 (89.5%) | 164/202 (81.2%) | 230/281 (81.9%) |
| Approval,  yes/total responses (%) | 57/60 (95%) | 18/19 (94.7%) | 183/202 (90.6%) | 258/281 (91.8%) |
| Treatment if APOE44,  yes/total responses (%) | 40/60 (66.7%) | 15/19 (78.9%) | 117/202 (57.9%) | 172/281 (61.2%) |
| Approval for APOE44,  yes/total responses (%) | 50/60 (83.3%) | 19/19 (100%) | 146/202 (72.3%) | 215/281 (76.5%) |

Supplementary Table: **Population numbers and survey responses regarding lecanemab treatment and approval stratified by region.** Abbreviations: DNG = Deutsches Netzwerk Gedächtnisambulanzen (German network of memory clinics); EADC = European Alzheimer’s Disease Consortium
